# Antifungal Activity, Biocompatibility, and Anti-Inflammatory Effects of Zerumbone-Enhanced Antimicrobial Photodynamic Inactivation in a Three-Dimensional Oral Tissue Model

**DOI:** 10.1021/acsomega.6c01470

**Published:** 2026-07-03

**Authors:** César Augusto Abreu-Pereira, Sarah Raquel De Annunzio, Ana Luíza Gorayb-Pereira, Paula Aboud Barbugli, Carlos Eduardo Vergani, Ana Cláudia Pavarina

**Affiliations:** Laboratory of Applied Microbiology—Department of Dental Materials and Prosthodontics, Faculdade de Odontologia de Araraquara, 153998 Universidade Estadual Paulista “Júlio de Mesquita Filho” (UNESP), Rua Humaitá, 1680, Araraquara, São Paulo 14803-901, Brazil

## Abstract

This study investigated the antifungal activity, biocompatibility, and anti-inflammatory effects of zerumbone (ZER; monocyclic sesquiterpene from the essential oil of ginger with antibiofilm activity) combined with antimicrobial photodynamic inactivation (aPDI) using a three-dimensional (3D) coculture model infected with *Candida albicans* biofilms fluconazole-sensitive (CaS; ATCC 90028) and fluconazole-resistant (CaR; ATCC 96901). For this purpose, a 3D coculture model involving two cell lines (fibroblasts and keratinocytes) was developed and infected with CaS and CaR biofilms. After biofilm establishment, treatments were performed using ZER (1172 or 2344 μM) and aPDI [ (PDZ;154 μM) and LED light (660 nm; 50 J/cm^2^; 44.5 mW/cm^2^)], either individually or in combination. Control groups included a conventional antifungal drug, a negative control (PBS), and a cell death-induced control (Triton X-100). Regarding uninfected 3D oral tissues, ZER (1172 μM) + aPDI exhibited minimal cytotoxicity by alamarBlue, with viability reduction under 18%. In the infected 3D coculture model, ZER (1172 μM) + aPDI resulted in reductions in viable colonies counting of 2.36 log_10_ for CaS and 2.15 log_10_ for CaR. Cell damage measured by lactate dehydrogenase release was 7.41% for CaS and 9.43% for CaR. The confocal laser scanning microscopy imaging supported these findings, suggesting that ZER + aPDI may impair the penetration of the fungal infection. Furthermore, ZER combined with aPDI reduced proinflammatory cytokines (IL-6 and IL-8) induced by *C. albicans* infection. Overall, evaluation of the 3D coculture model showed that the combination of ZER and aPDI was considered noncytotoxic and effective against fluconazole-resistant *C. albicans* biofilms. Therefore, this combination of approaches presents a promising strategy for combating resistant fungal infections, offering a potential alternative to conventional antifungal agents.

## Graphical abstract

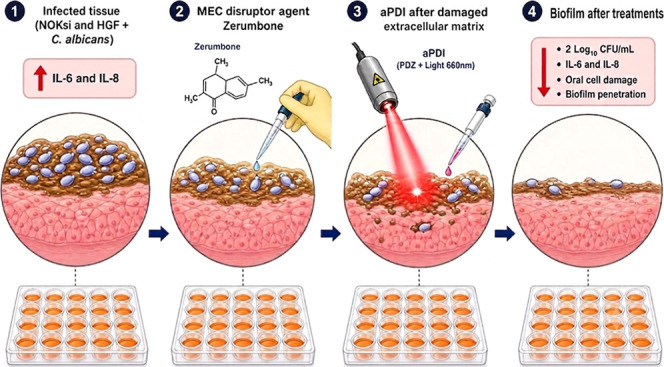

## 1 Introduction

Among the fungal species present in the oral cavity, *Candida albicans* is the most prevalent opportunistic pathogen and a major cause of nosocomial infections worldwide, mostly affecting immunocompromised individuals.^1^ Biofilms are structures strongly associated with *C. albicans* infections, conferring tolerance and resistance to conventional antifungal therapies and acting as a reservoir for recurrent infections.^2–4^ The clinical impact of *C. albicans* biofilm formation is correlated with increased virulence and higher patient mortality rates.^5^

Conventional antifungal therapies involve the local and/or systemic use of drugs from the azole class (fluconazole, voriconazole, and itraconazole), polyenes (amphotericin B and nystatin), and echinocandins (caspofungin, anidulafungin, and micafungin). These agents act by inhibiting vital fungal processes such as cell membrane synthesis.^6,7^ However, genetic alterations in microorganisms favor the development of resistance to these therapies by enabling adaptations in enzymatic pathways targeted by the drugs.^6^ Some of the resistance mechanisms in *C. albicans* are the upregulation of drug efflux pumps, differential target gene expression, the presence of an extracellular matrix (ECM) of the biofilm, persistent cells, and diverse stress responses.^8^ Thus, the conception of novel strategies of treatment is strongly suggested to effectively address infections caused by microorganisms that have developed resistance to conventional therapies.

Various compounds present in plant extracts exhibit a high content of active phytochemical such as antioxidant, anti-inflammatory, antiviral, antifungal, and antiproliferative properties.^9^ Zerumbone (ZER) is a monocyclic sesquiterpene present in the essential oil of ginger from the *Zingiber zerumbet* species.^10^ ZER is a compound that has shown promising activity against *C. albicans* biofilms,^11–13^ including the reduction of the ergosterol content, which is the main sterol component that acts on the structural characteristics of fungal filaments, affecting proper cell function, fluidity, and bioactivity of many membrane-bound regulatory enzymes.^14^

In a previous study, ZER at 256 μg/mL (1172 μM) reduced the fungal load of both fluconazole-sensitive and -resistant *C. albicans* biofilms about 37%.^12^ In addition, ZER reduced important ECM’s components: polysaccharides, proteins, and extracellular DNA.^12^ ZER might contribute to both the induction and modulation of reactive oxygen species (ROS) production, produced by various metabolic pathways (NADPH, mitochondria, oxidase, xanthine oxidase, nitric oxide synthase, cyclooxygenase, and enzymes), leading to cell inactivation and biofilm disruption.^15^

Antimicrobial photodynamic therapy (aPDI) is also an alternative treatment for inactivating *C. albicans* biofilms with efficacy proven in vitro,^16–18^ in vivo,^19–21^ and in clinical trials.^22,23^ aPDI also generates ROS, which induces cell inactivation through the excitation of a photosensitizer by a light source in the presence of oxygen.^24^ The improved effect of ZER and aPDI in the colony-count reductions in both fluconazole-sensitive and resistant *C. albicans* biofilm due to the prior action of ZER in weakening the ECM of *C. albicans* biofilms has been demonstrated.^25^

ROS are intercellular signaling molecules that regulate cellular processes; however, at very high levels, they can trigger inflammatory responses.^26^ These inflammatory responses are characterized by the production of proinflammatory cytokines, by several cell signaling pathways.^27^ In the inflammatory process induced by *C. albicans* biofilms, the production of proinflammatory cytokines [interleukins (IL)—IL-6, IL-8, and 1β] was observed^28,29^ Previously, it was reported that ZER suppressed the production of several inflammatory mediators by inhibiting the nuclear factor (NF-κB) pathway in cells of the human periodontal ligament.^30^ In aPDI, the production of inflammatory responses is intrinsically related to the type of photosensitizer and light parameters employed.^31^ Thus, it is necessary to evaluate the biocompatibility of ZER combined with aPDI to determine whether the treatment also affects mammalian cells.^26,32^

Although cell assays cultured in 2D models are still widely used to evaluate the cytotoxicity of novel therapeutic strategies, they fail to reproduce the dynamism and complexity of in vivo tissues.^33^ Another limitation is the lack of cellular diversity within these models, since a larger number of cell lines can better accurately reproduce the characteristics of tissues, producing more reliable results in in vitro assays.^34^ Thus, three-dimensional (3D) oral tissue models have been used in studies to evaluate the biocompatibility of various compounds, ensuring the reliability of results.^33,35,36^ Therefore, this study evaluated the biocompatibility, antifungal effect, and anti-inflammatory response of ZER and aPDI association in the treatment of *C. albicans* biofilms in 3D coculture models. For this purpose, two hypotheses were tested: H_0(null hypothesis)_: ZER + aPDI in 3D coculture model contaminated with *C. albicans* biofilm does not reduce the viable colony count of *C. albicans*, is not biocompatible, and does not reduce the levels of proinflammatory cytokines. H_1(alternative hypothesis)_: ZER + aPDI in 3D coculture model reduces the viable colony count of *C. albicans*, it is biocompatible, and reduces the proinflammatory cytokines.

## 2 Experimental Section

### 2.1 ZER Preparation

Stock solutions containing ZER (Sigma-Aldrich, St. Louis, MO, USA) were prepared by dissolving ZER crystals in 1 mL of dimethyl sulfoxide (DMSO—Sigma-Aldrich, St. Louis, MO, USA). Dilutions from the stock solution (10 mg/mL) was then conducted with sterilized water to obtain final concentrations of 293, 586, 1172, and 2344 μM, determined in a previous study.^12^ ZER’s chemical structure is present in Figure 1A.

**Figure 1:**
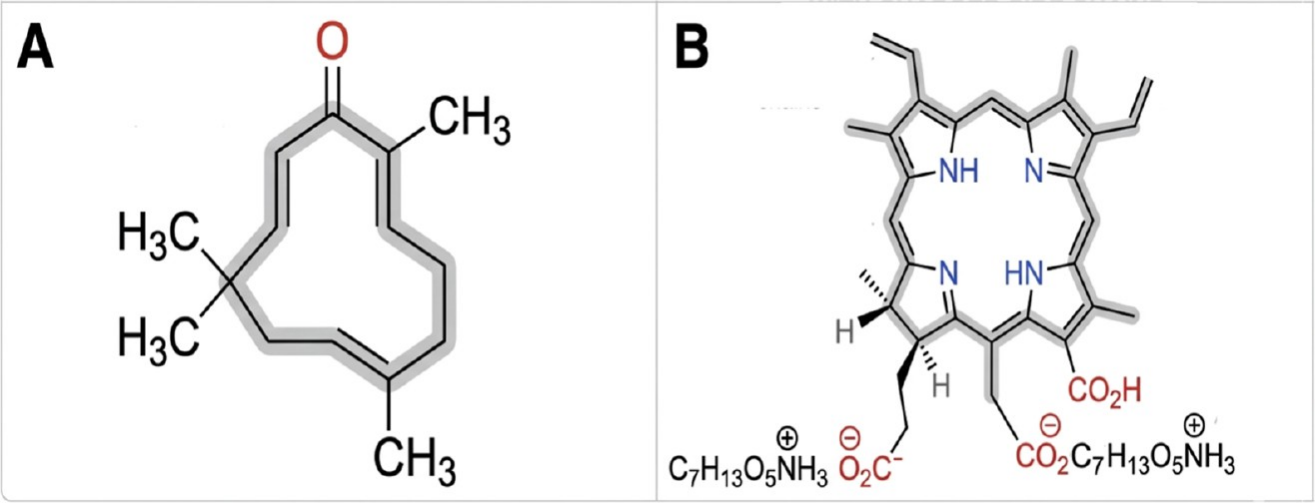
Chemical structures of ZER (A) and PDZ (B).

### 2.2 Photosensitizer Agent and Light Parameters

A bis-*N*-methylglucamine salt of chlorin e6 (Figure 1B) was used in this study as the photosensitizer, commercially known as photodithazine (PDZ; VETA-GRAND Co., Russia) at 154 μM.^37^ The absorption peak of PDZ is 660 nm. A red LED light was employed as the light source (660 nm; LXHL-PR09, Luxeon III Emitting, Lumileds Lighting, San Jose, California, USA) at a dose of 50 J/cm^2^ and an irradiance of 44.5 mW/cm^2^.^37^ The LED was equipped with a cooling system to prevent overheating. This system ensured that the tissues were not affected by possible temperature variations caused by the LED light.

### 2.3 Cell Culture Conditions

Human gingival fibroblast (HGF—Rio de Janeiro; code: 0089) and oral keratinocytes (NOK-si—supplied by Professor Carlos Rossa Jr., UNESP)^38^ were included in the present study. Dulbecco’s modified Eagle’s medium (DMEM—high glucose −4.5 g/L, Sigma, St. Louis, MO, USA) was used for the cell culture and supplemented with 10% (v/v) fetal bovine serum (FBS, Gibco, Life Technologies, USA), an antibiotic and antimycotic solution (penicillin G −28.061 μM, streptomycin −6.862 μM, and amphotericin B −27 μM) (Sigma-Aldrich, MO, USA), and l-glutamine (2.000 μM; Sigma-Aldrich, MO, USA). Cell cultures were maintained at 37 °C in a humidified atmosphere containing 5% CO_2_ and 80% humidity.

### 2.4 Biocompatibility of ZER in Monolayer (2D)

HGF and NOK-si cells were cultured in 75 cm^2^ flasks (Kasvi—Curitiba, PR, Brazil) at 70% confluence and were washed with sterile phosphate-buffered saline (PBS; 8 g NaCl, 1.44 g Na_2_HPO_4_, 0.2 g KCl, 1 L H_2_O, 0.24 g KH_2_PO_4_; pH 7.4), and detached using trypsin/EDTA solution [(0.05% v/v)/(0.53 mM); 7 min]. Following centrifugation (2.000 rpm; 5 min), 1 × 10^4^ cells/mL were dispensed into individual wells of 96-well dishes. The dishes were incubated for 24 h at 37 °C with 5% CO_2_. After this period, the medium was removed from the wells, and 200 μL of ZER solutions (293, 586, 1172, or 2344 μM) were added to each well. Control groups included 200 μL of 1× PBS (negative control), 200 μL of a nystatin solution (Sigma-Aldrich, MO, USA) at a concentration of 270 μM (positive control), and Triton X-100 0.9% (death control). The vehicle (2% DMSO) in which ZER was diluted was also evaluated. Treatment was performed for 20 min.^12^ Subsequently, the treatment solutions were removed, and 200 μL of DMEM and 20 μL of alamarBlue solution (Thermo Fisher Scientific, MA, USA) were added to each well. After 16 h of incubation, the fluorescence intensity was measured using a fluorescence reader (FLUOstar Omega, BMG Labtech, Cary, NC, USA; Ex: 544–10 nm; Em: 590–10 nm).^38,39^ Three independent experiments (biological replicates; *n* = 3) were performed in quadruplicate (technical replicates; *n* = 4), resulting in a total sample size of *n* = 12 per group.

### 2.5 Development of 3D Cell Coculture Model and Treatment

The 3D cell coculture model was developed using cells between passages 3 and 10 that were cultured in 75 cm^2^ flasks until reaching 70% confluence (Kasvi—Curitiba, PR, Brazil). After washing the cells with PBS, trypsin solution was used for cell detachment, and they were centrifuged (2.000 rpm/5 min). To prepare the dermal layer, 2.5 mL of DMEM (high glucose −4.5 g/L) without antibiotic and antimycotic solution, 500 μL of PBS, and 2 mL of type I collagen (from the tail of Wistar rats) were used. This solution was neutralized using 30 μL of 1 M NaOH, and 2.5 mL of the HGF cell suspension at 0.3 × 10^6^ cells/mL was added. Afterward, 500 μL was transferred to 24-well plates, and the samples were incubated to promote dermal layer polymerization for 2 h. After this period, 200 μL of the NOK-si cell suspension (2.1 × 10^6^ cells/mL), prepared in DMEM medium, without antibiotic and antimycotic solution, was dispensed into each well over the dermal layers, and the samples were incubated for 24 h for epithelial layer establishment.^36^ The treatments were performed according to the protocol outlined in Table 1. Three independent experiments (biological replicates; *n* = 3) were performed in quadruplicate (technical replicates; *n* = 4), resulting in a total sample size of *n* = 12 per group.

**Table 1:** Treatments Carried out on 3D Cell Coculture Model

control	PBS solution (500 μL)
ZER 1172 μM	ZER 1172 μM (500 μL) for 20 min
ZER 2344 μM	ZER 2344 μM (500 μL) for 20 min
light	Red LED (20 min, 50 J/cm^2^)
ZER 1172 μM + Light	ZER 1172 μM (500 μL) for 20 min, and irradiation with a red LED (20 min, 50 J/cm^2^)
ZER 2344 μM + Light	ZER 2344 μM (500 μL) for 20 min, and irradiation with a red LED (20 min, 50 J/cm^2^)
aPDI	PDZ (154 μM; 500 μL) was added; the plate was incubated for 20 min and irradiated with a red LED (20 min, 50 J/cm^2^)
ZER 1172 μM + aPDI	ZER at 1172 μM (500 μL) for 20 min. Subsequently, the aPDI was performed
ZER 2344 μM + aPDI	ZER at 2344 μM (500 μL) for 20 min. Subsequently, the aPDI was performed
nystatin (NYS)	NYS 270 μM (500 μL) for 20 min
death control	Triton X-100 0.9% (500 μL) for 20 min
2% DMSO	2% DMSO (500 μL) for 20 min

### 2.6 Biocompatibility of Noninfected 3D Cell Coculture Model by alamarBlue

After the treatments, the 3D cell coculture model was resuspended in 300 μL of DMEM followed by incubation (24 h). Then, 30 μL of alamarBlue (Thermo Fisher Scientific, MA, USA) was added to the wells and the plate was incubated at 37 °C with 5% CO_2_ for 16 h. After incubation, a 100 μL aliquot from each well was transferred to a 96-well plate in triplicate, and the fluorescence intensity was measured (FLUOstar Omega, BMG Labtech, Cary, NC, USA; Ex.: 544–10 nm; Em.: 590–10 nm).^38,40^

### 2.7 Infection of 3D Cell Coculture Model with *C. albicans* Biofilm and Treatment

For 3D cell coculture infection, two reference strains of sensitive *C. albicans* (ATCC 90028; CaS) and fluconazole-resistant *C. albicans* (ATCC 96901; CaR) were employed. The reactivation of the strains was conducted on Sabouraud Dextrose Agar (SDA; KASVI, Paraná, Brazil) Petri dishes with chloramphenicol supplementation (0.1 g/L) for 24 h at 37 °C. Then, five colonies from each strain were incubated in 5 mL of Yeast Nitrogen Base (YNB; BD Difco, CA, USA) with glucose supplementation (100 mM) (preinoculum). Following 16 h of incubation, a dilution of the preinoculum was performed in fresh YNB medium (1:20) and incubated at 37 °C for an additional 8 h (inoculum). The resulting inoculum was then washed twice with 1× PBS and centrifuged (5000 rpm; 10 min; 4 °C). The pellets were resuspended in RPMI-1640 medium supplemented with 2 mM l-glutamine and sodium bicarbonate (2 g/L). The inoculum was adjusted to a concentration of 3 × 10^4^ CFU/mL.^36^

For 3D cell coculture infection, the DMEM medium was removed and *C. albicans* cell suspension (resuspended in RPMI, without antibiotics and antimycotics) was added onto each 3D cell coculture (100 μL), and the dishes were incubated (37 °C with 5% CO_2_ for 24 h) for biofilm formation.^36^ After 24 h, treatments were performed, as described in Table 1. Three independent experiments (biological replicates; *n* = 3) were performed in quadruplicate (technical replicates; *n* = 4), resulting in a total sample size of *n* = 12 per group.

### 2.8 Cell Damage of Infected 3D Cell Coculture Model by Lactate Dehydrogenase Release

Twenty-four hours after the treatment, 500 μL of DMEM medium was collected from each 3D coculture model. To assess epithelial cell damage in the infected 3D coculture model, LDH levels were quantified in the surrounding medium. Specifically, LDH release into the maintenance medium of the infected 3D coculture oral tissues was measured 24 h after infection. LDH activity was subsequently measured by fluorescence at excitation and emission wavelengths of 540 and 590 nm, respectively. LDH release was performed using the CytoTox-ONE kit (G7890-Promega, Madison, Wisconsin, USA) according to the manufacturer’s instructions. For this, 50 μL of supernatant aliquot from each 3D cell coculture was collected and transferred in quadruplicate to a 96-well plate. Then, 50 μL of the CytoTox-one reagent was added on each well and after 10 min the fluorescence intensity was measured (FLUOstar Omega, BMG Labtech, Carywas, NC, USA; Ex: 544–10 nm; Em: 590–10 nm).^36,41^

### 2.9 Antifungal Effect of ZER Associated with aPDI

The antifungal efficacy of the treatments was performed by colony-forming units per milliliter (CFU/mL) counting. Briefly, the infected 3D coculture was collected, transferred to a microtube with 1 mL of PBS, and vortexed for 5 min to dissociate the cells from the 3D cell coculture model. Subsequently, 100 μL of the resulting suspension was serially diluted with PBS (1:1.000), and 20 μL was spread onto SDA Petri dishes for each dilution. These dishes were incubated for 24 h (37 °C), and the number of viable cells was enumerated, and the CFU/mL was calculated.^37^ Following this step, the synergistic effect between ZER and aPDI was calculated using the Bliss independence model. The synergy scores will be categorized as follows: S < 0-antagonistic effect; S = 0-additive effect; S > 0 = Synergism.^42^

### 2.10 Cytometric Bead Array of Infected 3D Cell Coculture Model

Twenty-four hours after treatments, 500 μL of supernatant was collected from each well containing the 3D infected model. Cytokine quantification was performed using the BD Biosciences Human Cytometric Bead Array (CBA) kit for human inflammatory cytokines (San Jose, CA, USA). Briefly, the provided lyophilized human inflammatory cytokine standards were reconstituted with 2 mL of the kit’s Assay Diluent. A serial dilution of these standards was conducted. The negative control consisted solely of the BD Biosciences Assay Diluent. Following reconstitution and dilution, a bead mixture containing specific capture antibodies was prepared following the manufacturer’s protocol. The flow cytometry analysis was conducted with 50 μL of this mixed capture, which were added to microtubes designated for each point of the standard curve combined with the experimental samples. Then, 50 μL of each serially diluted human inflammatory cytokine standard was transferred to the respective tubes from the curve, and 50 μL of each experimental sample supernatant was added to their corresponding tubes. Subsequently, 50 μL of the BD Biosciences human inflammatory cytokine phycoerythrin was added to all tubes. These tubes were then incubated for 3 h in the dark. Following incubation, 1 mL of the kit’s wash buffer was added to each tube, and the tubes were centrifuged at 5000 rpm for 5 min. The supernatant was carefully removed, and 300 μL of the BD Biosciences wash buffer was added to each tube. Data acquisition was performed using a BD Biosciences FACS Aria Fusion II Flow Cytometer (San Jose, CA, USA). The acquired data were analyzed using FCAP Array software version 3.1 (BD Biosciences).^36^

### 2.11 Confocal Laser Scanning Microscopy

To characterize the 3D coculture model, new tissues were formed, and confocal laser scanning microscopy (CLSM) assays were performed using a confocal fluorescence microscope LSM 800 Carl Zeiss with Zen Blue Software vs 2.3 (Carl Zeiss, Jena, Germany). CLSM was used to qualitatively visualize the architecture and viability of the 3D cell coculture model, allowing detailed observation of fungal invasion, cellular integrity, and spatial distribution of the cells after treatment. For this, 3D cell cocultures were washed with PBS, and 500 μL of propidium iodide (PI) solution (1:1000 dilution) (Thermo Fisher Scientific, MA, USA) was incubated with the samples for 10 min. Next, the 3D cell cocultures were washed with 1× PBS to remove unspecific PI binding. Subsequently, 300 μL of 4% (v/v) paraformaldehyde was added, and the cocultures were incubated (20 min; 37 °C). The 3D models were washed with 1× PBS, and 300 μL of 0.1% (v/v) Triton X-100 solution was added and incubated for 10 min. Then, the 3D models were washed twice and stained with ActinGreen 488 ReadyProbes reagent (Thermo Fisher Scientific, MA, USA) for 30 min. Finally, the wells were stained with Calcofluor White (Sigma-Aldrich, MO, USA) for 10 min. Then, the CLSM of the 3D cell cocultures was conducted. For imaging acquisition, 405 nm, 488, and 561 nm lasers were used in a 10× objective.^36^

### 2.12 Statistical Analysis

The data from independent experiments were pooled for analysis. First, the data were subjected to descriptive statistics to determine mean, standard deviations, and possible outliers. Then, the tests were conducted to assess normal distribution and homoscedasticity, by Shapiro–Wilk’s and Levene’s test, respectively. All independent variables met the normality assumption (*p* > 0.05). The one-way ANOVA was performed, following Tukey’s post hoc test for homoscedastic data [cytotoxicity 2D, cytotoxicity in noninfected 3D coculture, cell damage in infected 3D coculture (CaS and CaR), CFU/mL (CaS), and cytokine production in infected 3D coculture] and Games–Howell’s post hoc test for heteroscedastic data [CFU/mL (CaR)]. The IBM SPSS Statistics (version 21, IBM Corp.) software was used in all analysis with 5% of significance.

## 3 Results

### 3.1 Biocompatibility of ZER in Monolayer (2D)

Following post hoc analyses, it was observed for both NOK-si cells (Tukey’s test; Figure 2A) and HGF cells (Games–Howell’s test; Figure 2B) that the experimental groups ZER 293, ZER 586, and ZER 1172 were statistically similar in cell viability compared to the control group (*p* ≥ 0.051) and were statistically similar (*p* ≥ 0.296) to each other. As well as the NYS and 2% DMSO groups showed no statistically significant difference (*p* ≥ 0.694) from the control group and were statistically similar to each other (*p* ≥ 0.752).

**Figure 2:**
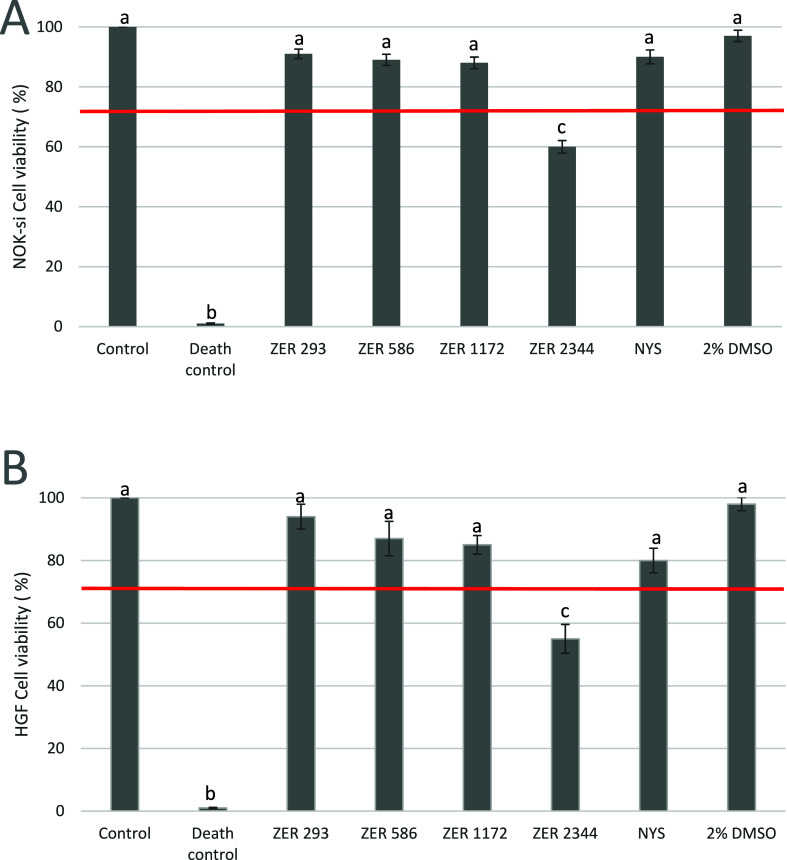
Percentage of cell viability of NOK-si cell (A) and HGF cell (B) monolayers after the treatments. The red line point corresponds to the acceptable reduction limit (30%) of cell death. Significant differences are represented by different letters, as determined by the Tukey post hoc test (*p* < 0.05).

In contrast, the ZER 2344 group induced a statistically significant reduction in the cellular viability of both NOK-si and HGF cells (42.5%) when compared to the control group (*p* ≤ 0.002) and to all other treatment groups (*p* ≤ 0.005). The group corresponding to the death control showed a reduction in cells (NOK-si and HGF) of more than 99% and was statistically different from all other groups (*p* < 0.0001).

### 3.2 Biocompatibility of Treatments in a Noninfected 3D Cell Coculture Model Assessed by alamarBlue

For the uninfected 3D cell coculture, following Games–Howell’s post hoc test, it was observed that only the ZER 1172 μM and 2% DMSO groups were statistically similar (p = 1.000) to the control group. The NYS, light, ZER 1172 μM + light, aPDI, and ZER 1172 μM + aPDI groups were statistically different from the control group (*p* < 0.001); although a decrease in the 3D coculture cell viability was observed, it remained within biocompatible levels (<18%) (ISO, 2009).^40^ The ZER 2344 μM, ZER 2344 μM + light, and ZER 2344 μM + aPDI groups were statistically different from the control group (*p* < 0.001) and showed a reduction in viable cells exceeding 30%. These data are shown in Figure 3.

**Figure 3:**
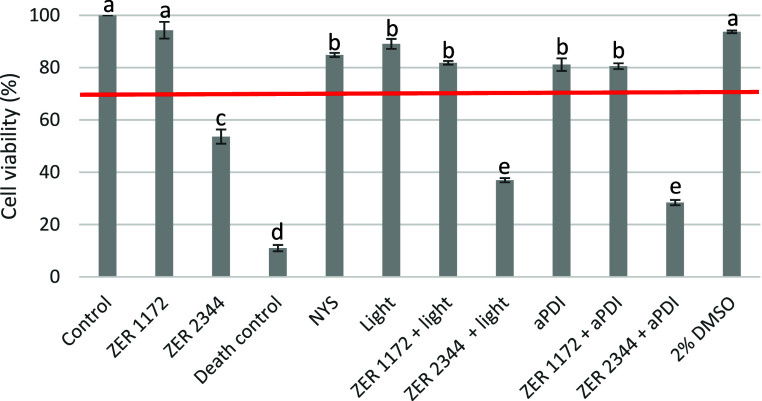
Biocompatibility of treatments in a noninfected 3D cell coculture model assessed by alamarBlue. The red line points to the acceptable reduction limit (30%) of cell death. Significant differences are represented by different letters, as determined by the Tukey post hoc test (*p* < 0.05).

### 3.3 Cell Damage by Lactate Dehydrogenase Release and Antifungal Activity of the Treatments

#### 3.3.1 3D Cell Coculture Infected with Fluconazole-Sensitive *C. albicans* (CaS)

In the LDH release quantification of 3D cell coculture infected with CaS (Figure 4A), a statistically significant difference (*p* < 0.001) was observed between the treatment groups and the death control group. The ZER 1172, ZER 2344, ZER 1172+ light, 2% DMSO, ZER 2344 + light, aPDI, ZER 1172 + aPDI, and ZER 2344 + aPDI groups showed minor LDH release (<13.8%) with no statistically significant differences among them (*p* ≥ 0.166) and values comparable to the noninfected control. The infected group was statistically different (p = 0.047), presenting the second largest cell damage (highest LDH release). The light group showed a superior liberation of LDH, and it was statistically similar to the infected control (p = 0.052) and different from other groups (*p* ≤ 0.042). The NYS group showed intermediate values of cell damage, and it was statistically different from the other groups (*p* ≤ 0.022). The noninfected control group (*p* ≥ 0.098) was different from the death control and the infected control groups (*p* ≤ 0.038). The noninfected control group was similar to those treated with ZER, aPDI, and ZER + aPDI, indicating that the proposed treatment inhibits the ability of *C. albicans* to cause damage and promote LDH release.

**Figure 4:**
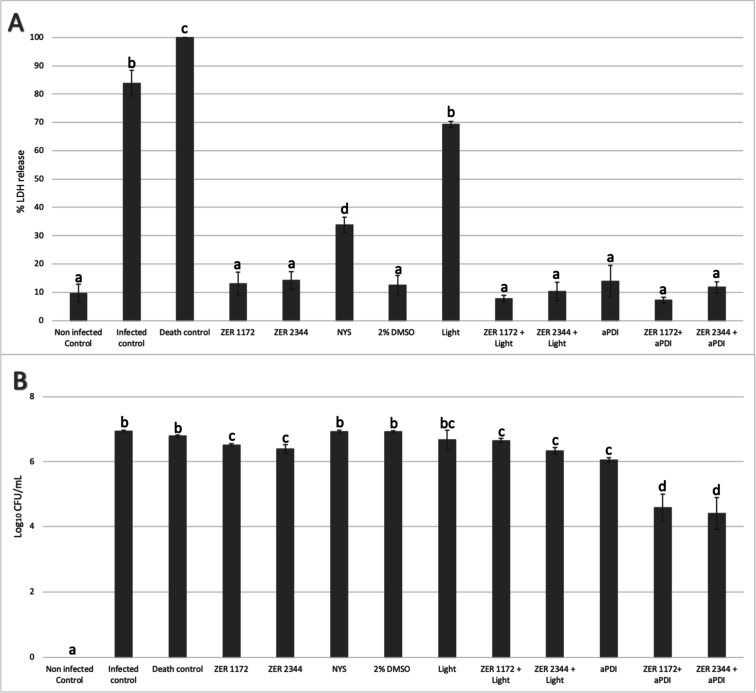
Means and standard deviations of lactate dehydrogenase (LDH) release (A) and fungal load (CFU/mL) (B) in 3D cell coculture infected with fluconazole-sensitive *C. albicans*. Significant differences are represented by different letters, as determined by the Games–Howell post hoc test (A and B; *p* < 0.05).

In the results of CaS colony count (CFU/mL) (Figure 4B), a statistically significant difference (*p* < 0.001) was observed among the treatment groups. The ZER 1172+ aPDI and ZER 2344+ aPDI groups showed the greatest reductions in fungal load, exhibiting a statistically significant difference from the infected control group (*p* ≤ 0.022) and being similar to each other (p = 0.056). The ZER 1172, ZER 2344, ZER 1172+ light, ZER 2344 + light, and aPDI groups also showed statistically significant reductions in fungal load compared to the control group (*p* ≤ 0.039), albeit to a smaller extent. The remaining groups were similar (*p* ≥ 0.063) to the infected control group.

#### 3.3.2 3D Cell Coculture Infected with Fluconazole-Resistant *C. albicans* (CaR)

In the LDH release quantification of 3D cell coculture infected with CaR (Figure 5A), a statistically significant difference (*p* < 0.001) was observed between the treatment groups and the infected control group. The ZER 1172 and ZER 2344 groups associated with aPDI or light showed the smallest reductions (<12.1%) in LDH release, being statistically similar to each other (*p* ≥ 0.418) and to the uninfected control group (*p* ≥ 0.572), and different from the death control and infected control groups (*p* ≤ 0.002). The ZER 1172, ZER 2344, 2% DMSO, and aPDI groups also showed a reduction in LDH release; however, they were statistically different from the groups where ZER was associated with aPDI (*p* ≤ 0.014). The NYS and light groups were similar to each other (p = 0.108) and different from all other groups (*p* ≤ 0.012).

**Figure 5:**
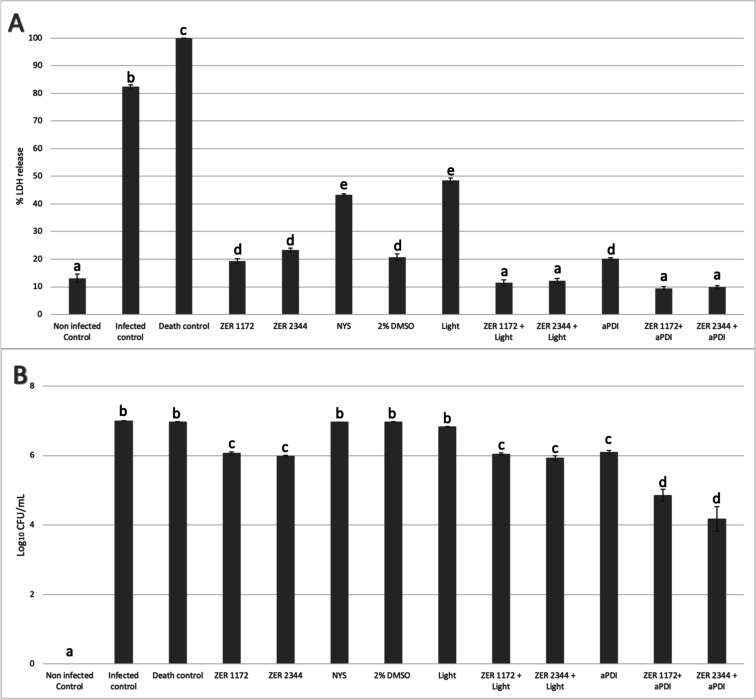
Means and standard deviations of lactate dehydrogenase (LDH) release (A) and antifungal activity (CFU/mL) (B) in 3D cell coculture infected with fluconazole-resistant *C. albicans*. Significant differences are represented by different letters, as determined by the Tukey post hoc test (A) and Games–Howell post hoc test (B) (*p* < 0.05).

The results of fungal load (CFU/mL) of CaR after the treatments (Figure 5B) showed a statistically significant difference among the treatment groups (*p* < 0.001). The ZER 1172 + aPDI and ZER 2344 + aPDI groups were similar to each other (p = 0.606) and showed the greatest reductions in fungal load, being statistically different from the infected control group (*p* < 0.001). The ZER 1172, ZER 2344, ZER 1172+light, ZER 2344+light, and aPDI groups also showed statistically significant reductions in fungal load compared to the control group (*p* ≤ 0.009), albeit to a lesser extent. The remaining groups were similar (*p* ≥ 0.103) to the infected control group.

### 3.4 Bliss Independence Model

Drug interaction analysis using the Bliss independence model^42^ revealed synergistic behavior (S > 0) in both strains tested (Table 2), regardless of their resistance profiles. For the CaS biofilm, the observed effect exceeded the expected effect by 16.04% (ZER 1172 + aPDI) and 15.25% (ZER 2344 + aPDI). Similarly, a synergistic effect was observed for the CaR biofilm, with an increase in antimicrobial efficacy of 6.04% (ZER 1172 + aPDI) and 14.75% (ZER 2344 + aPDI).

**Table 2:** Bliss Independence Model for Analyzing the Synergistic Effect of the Drug Combination (ZER and aPDI)^a^

biofilm	ZER concentration	ZER effect (*E*_Zer_)	aPDI effect (*E*_aPDI_)	expected effect (*E*_expZER+aPDI_)	observed effect (E_obsZER+aPDI_)	synergy score (S)
CaS	1172	0.0605	0.1268	0.1796	0.3400	^**+**^ **0.1604**
	2344	0.0992	0.1268	0.2134	0.3659	^**+**^ **0.1525**
CaR	1172	0.1342	0.1300	0.2468	0.3071	^**+**^ **0.0604**
	2344	0.1457	0.1300	0.2568	0.4042	^**+**^ **0.1475**

a The synergy scores (S) were categorized as follows: S < 0-antagonistic effect; S = 0-additive effect; S > 0 = synergism.^42^

### 3.5 Cytometric Bead Array of Infected 3D Oral Tissues

Cytokine production by 3D cell coculture infected with CaS and CaR biofilms after the treatments was assessed using flow cytometry (Figure 6). The ZER 1172 + aPDI and ZER 2344 + aPDI groups exhibited the most significant reduction in the production of IL-6 and IL-8 compared with the infected control group (*p* < 0.001), which did not receive treatment, regardless of the strain evaluated (CaS or CaR). These groups were similar to each other (*p* ≥ 0.892) and different from all other groups (*p* ≤ 0.012). The ZER 1172, ZER 2344, aPDI, ZER 1172 + Light, and ZER 2344 + Light groups also demonstrated a reduction in IL-6 and IL-8 levels compared to the infected control group (*p* ≤ 0.003), with a decrease of approximately 80% or more. The NYS and Light groups showed a smaller, but still statistically significant (*p* ≤ 0.004), reduction in IL-6 and IL-8 compared to the infected control group. Quantification IL-12p70, TNF, IL-10, and IL-1β were also performed but did not reveal any detection across all evaluated groups.

**Figure 6:**
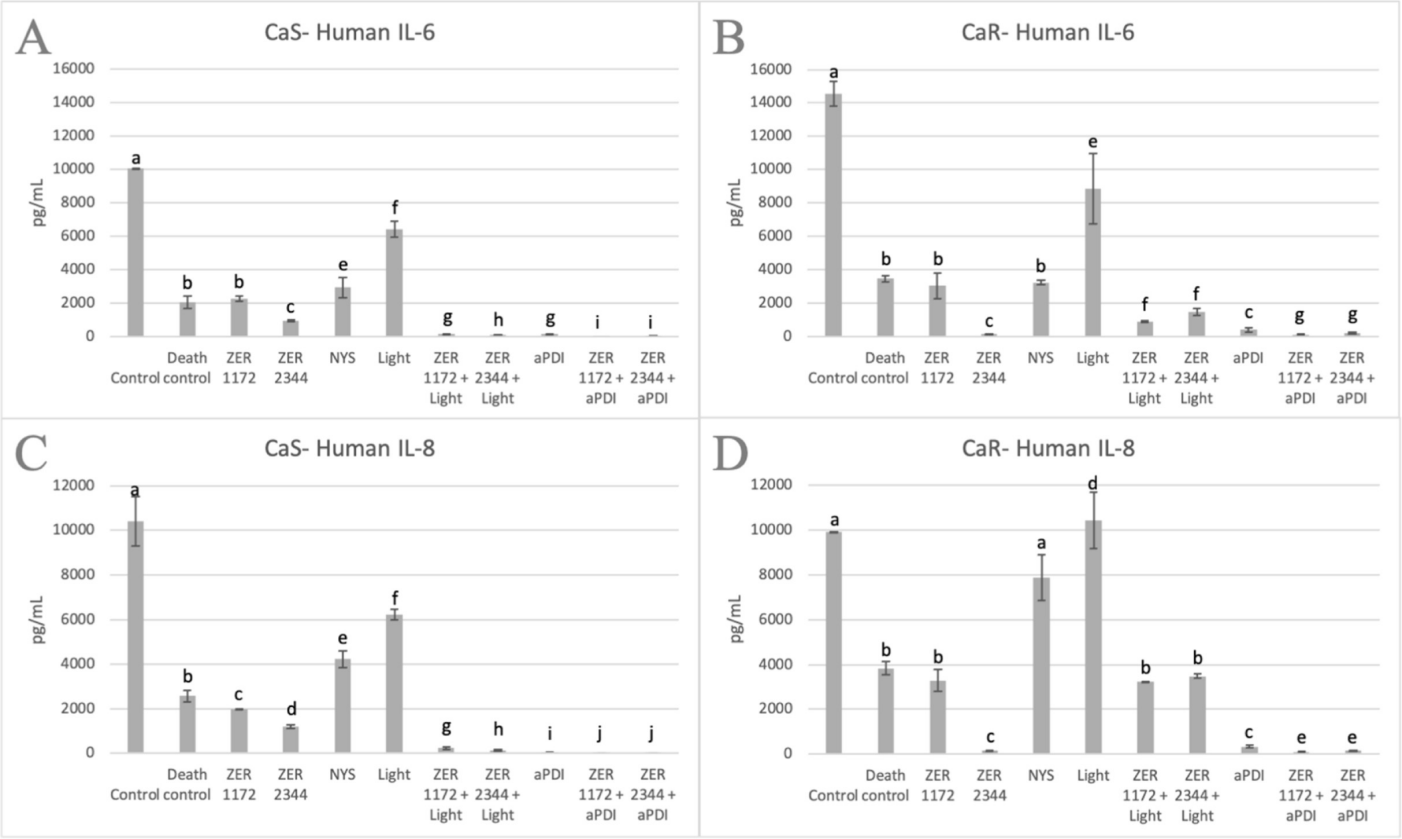
Comparisons of means and standard deviations in the cytokine (IL-6 and IL-8) production in 3D coculture infected with fluconazole-sensitive (A and C) and -resistant (B and D) *C. albicans* following treatment. Significant differences are represented by different letters, as determined by the Tukey post hoc test (*p* < 0.05).

### 3.6 Confocal Laser Scanning Microscopy

CLSM qualitative observations of the 3D cell coculture model infected with *C. albicans* corroborated with CFU/mL findings, suggesting that the ZER + aPDI combination exhibited superior antibiofilm activity compared to ZER or aPDI alone in both the CaS (Figure 7) and CaR biofilms (Figure 8). ZER + aPDI groups reduced the *C. albicans* biofilm (blue area). A more intact epithelial layer (green area) and fewer dead cells (red spots) were evident. The ZER 1172, ZER 2344, ZER 1172 + Light, ZER 2344 + Light, aPDI, ZER 1172 + aPDI, and ZER 2344 + aPDI groups showed a limited penetration (yellow arrows) of the *C. albicans* infection, which was limited to the epithelial surface of the 3D cell coculture infected with CaS and CaR. Conversely, in all groups without ZER or aPDI, the fungal infection visually appeared to penetrate the tissue layers (red arrows). The group treated with aPDI only displayed a pattern comparable to that of the ZER-treated groups.

**Figure 7:**
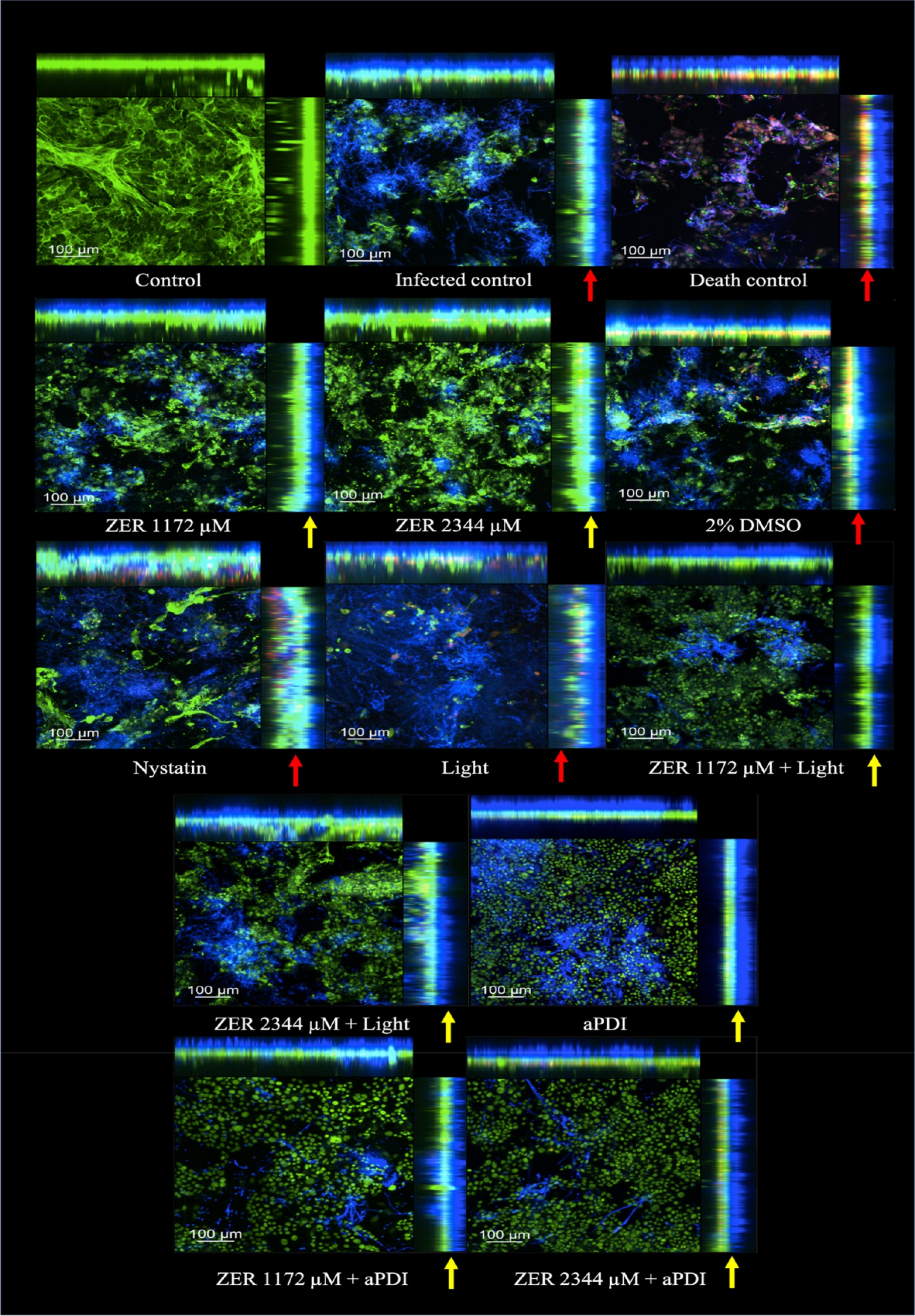
Confocal laser scanning microscopy (CLSM) images of 3D cell coculture model infected with fluconazole-sensitive *C. albicans* (CaS) after treatment. Green: cytoskeleton (stained by ActinGreen); red: dead cells (stained with PI); blue: CaS biofilms (stained by CalcoFluoR White). Yellow arrows indicate minor penetration of the infection, while red arrows indicate major penetration into the 3D oral tissue.

**Figure 8:**
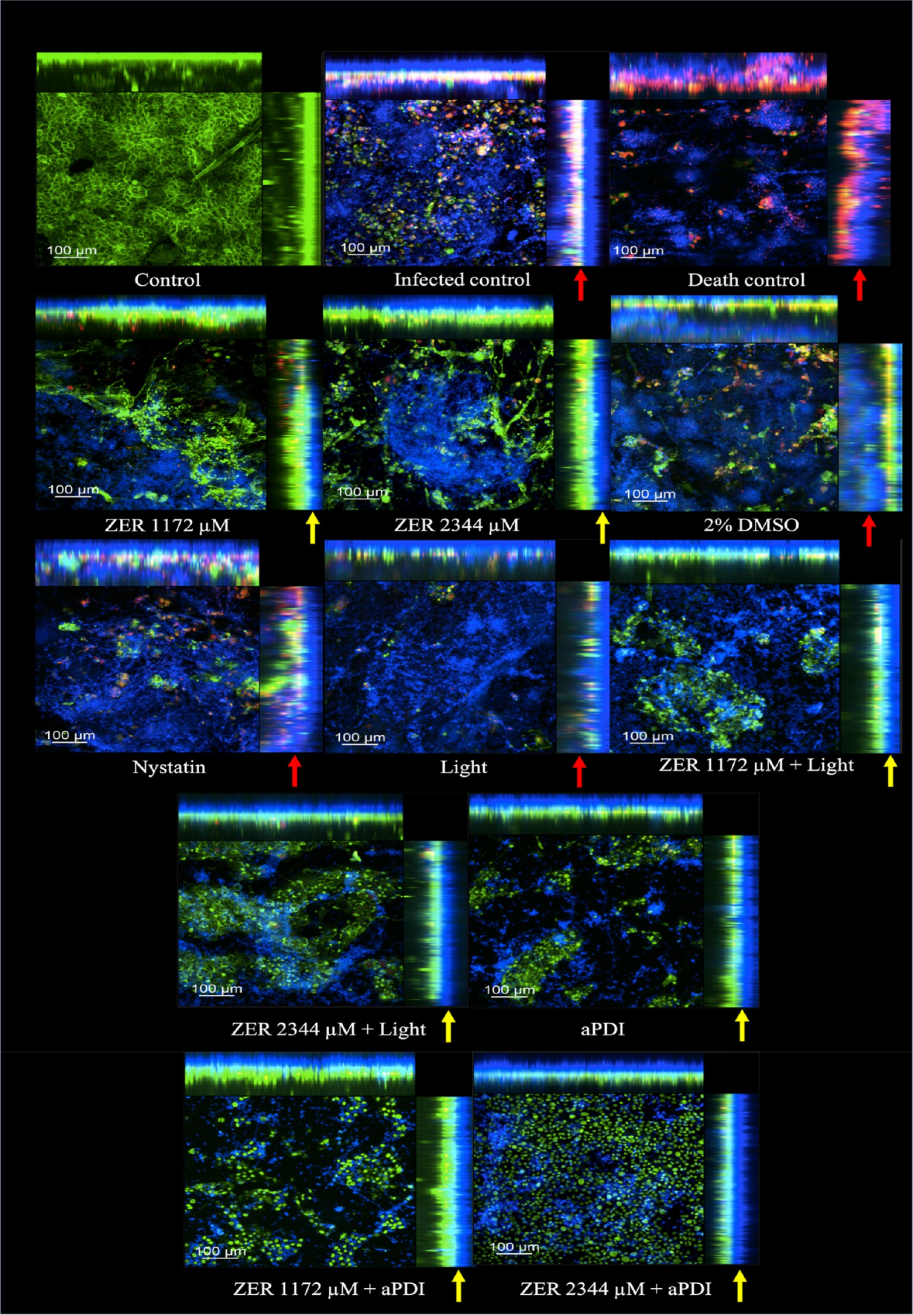
Confocal laser scanning microscopy (CLSM) images of 3D cell coculture infected with fluconazole-resistant *C. albicans* (CaR) after treatment. Green: cytoskeleton (stained by ActinGreen); red: dead cells (stained by PI); blue: CaR biofilms (stained by CalcoFluoR White). Yellow arrows indicate minor penetration of the infection, while red arrows indicate major penetration into the 3D oral tissue.

## 4 Discussion

The 3D coculture model is an innovative approach for studying biofilm formation and inhibition under conditions that mimic in vivo biological environment. Unlike traditional monoculture systems, 3D coculture models involve simultaneous interaction between different cell types, enabling the investigation of microbial behavior and host cell responses in a more complex and physiologically representative microenvironment.^43^ In biofilm research, 3D coculture model is particularly valuable, as it allows the analysis of how microorganisms adhere to, colonize, and persist on living biological surfaces.^43^ Furthermore, it allows the assessment of the efficacy of antimicrobial or therapeutic strategies under conditions that take into account pathogen and host interactions. This study evaluated the biocompatibility, antifungal activity, and anti-inflammatory effect of the combination of ZER with aPDI for treatment of *C. albicans* biofilms in a 3D coculture model. The inflammatory response induced by *C. albicans* biofilms was also appraised. In the present study, *C. albicans* cells were able to invade the 3D coculture model, corroborating with previous studies.^36,41^ The biocompatibility assay in the noninfected 3D coculture demonstrated that ZER combined with aPDI resulted in a mild reduction of less than 18% in cell viability, as measured by the alamarBlue assay, indicating good cytocompatibility. In the 3D coculture model infected with *C. albicans*, the combination of ZER at 1172 μM with aPDI also reduced cell damage (measured by LDH release). Moreover, the ZER + aPDI combination effectively reduced the fungal load by more than 2 log_10_ CFU/mL. Thus, the progression of infection to deeper layers of the 3D coculture models was attenuated. As a result, this intervention may have decreased the proinflammatory response induced by *C. albicans* by reducing interleukin levels, particularly IL-6 and IL-8, suggesting an anti-inflammatory effect.

Evaluating the cytotoxicity of novel therapeutic compounds is a critical step in preclinical development. In this study, the biocompatibility of the treatments was assessed using the alamarBlue assay. The noninfected 3D coculture model treated with ZER (ZER 1172 μM) followed by aPDI did not exhibit cytotoxic effects, showing a reduction in cell viability of less than 13.8%. This decrease is within the range considered acceptable according to ISO 10993-5 guidelines,^40^ which define a viability reduction below 30% as noncytotoxic. The positive control (NYS) and 2% DMSO (vehicle control) also showed noncytotoxic behavior. These findings support the potential safety of the ZER + aPDI combination for therapeutic application. The biocompatibility observed in the 3D coculture models in the present study is consistent with findings from previous studies performed in 2D models that had already confirmed the noncytotoxic behavior of ZER.^44,45^ It was demonstrated that ZER exhibited no significant cytotoxicity on Vero cells at concentrations up to 100 μg/mL, maintaining cell viability above 87% after 48 h of treatment.^44^ However, ZER at 2344 μM was considered cytotoxic, causing approximately a 40% reduction in oral cell viability. However, it is important to consider that mammalian cells tend to exhibit greater susceptibility compared in vivo models due to their contact with the biomolecules without physiological barriers influencing them.^46^

In the infected 3D coculture model, treatment with ZER (1172 μM) previously to aPDI resulted in 2.36 log_10_ and 2.15 log_10_ reductions in the fungal load of CaS and CaR biofilm, respectively. On the other hand, NYS (conventional antifungal) did not show antimicrobial activity. ZER’s concentrations used in the present study were determined by minimum inhibitory concentration (MIC_50_) and minimum fungicidal concentration (MFC) established in a previous study.^12^ CLSM images supported that the ZER + aPDI treatment exhibited superior antifungal activity and may be involved in the prevention and the penetration of CaS and CaR biofilms into the 3D coculture model. Analysis using the Bliss independence model^42^ also demonstrated a synergistic interaction (S > 0) between ZER (1172 or 2344) and aPDI, suggesting that the combination potentiates the antimicrobial effect by up to 15% compared to the expected independent action. These results are consistent with previous studies evaluating the efficacy of ZER combined with aPDI against *C. albicans* biofilms, showing reductions of 2.43 log_10_ in fungal load for the CaS biofilm and 2.04 log_10_ for the CaR biofilm, thereby demonstrating the effective action of ZER + aPDI in this regard.^25^ ZER is a terpenoid compound with the ability to inhibit *C. albicans* adhesion.^11^ The antifungal activity of terpenes is attributed to their ability to penetrate the fungal cell wall and incorporate into the lipid bilayer, thereby disrupting lipid organization and altering membrane structure.^47^ The variations in the fluidity and the permeability of the cell membrane can lead to cell wall disruption, reducing adhesion to host surfaces and inducing several consequences, including disturbance of the cytoplasmic membrane, leakage of intracellular contents, cytoplasmic coagulation, and cell lysis.^48^ In a previous study, the isolated application of ZER (1172 μM) reduced *C. albicans* biofilms. Specifically, CaS exhibited a decrease of 0.47 log_10_, whereas CaR and the two clinical isolates showed reductions of 1.24 log_10_ and 1.10 log_10_, respectively.^25^ The isolated application of ZER promoted a smaller decrease in biofilm viability and also prevented the penetration of *C. albicans* into the 3D coculture model, however, having lower antifungal efficacy compared to the ZER + aPDI treatment. The enhanced efficacy of the combination of ZER with aPDI against *C. albicans* biofilms stems from a complementary and multifaceted action: ZER disorganizes the extracellular biofilm matrix,^12^ which may compromise fungal membrane permeability,^47^ facilitating the photosensitizer penetration and increasing cellular vulnerability. ZER is lipophilic and can incorporate into the lipid bilayer of the fungal cell membrane, altering its fluidity and permeability.^44^ The compromised cell membrane makes the fungal cell more vulnerable to aPDI -induced oxidative damage.^24^ These findings indicate that ZER may disrupt the structural components of *C. albicans* biofilms, thereby facilitating the diffusion of PDZ and light into deeper biofilm layers, enhancing the overall effectiveness of photodynamic treatment.

Morphological analyses of the 3D coculture model revealed significant disruption of the epithelial layer following *C. albicans* infection and hyphae penetration, which is consistent with previous observations in similar full-thickness 3D coculture models.^36,41,49^ The cell damage was further confirmed by LDH release, which increased significantly in the 3D coculture model infected by *C. albicans*.^41,42^ Cataldi et al.^50^ demonstrated that a complex biofilm structure, comprising blastospores, pseudohyphae, and hyphae cells, increases the overall biofilm surface area, thereby enhancing adhesion to host cell surfaces and contributing to pathogenicity. The ability of *C. albicans* to adhere and to invade host tissues is linked with the secretion of hydrolytic enzymes such as proteases and phospholipases. As shown by CLSM, ZER treatment followed by aPDI demonstrated significant antibiofilm activity, limiting the invasive potential of *C. albicans* into the 3D coculture oral model. These observations corroborated the reductions in fungal load, demonstrating that the ZER + aPDI combination exhibited superior antibiofilm activity compared with either ZER or aPDI alone in both CaS (Figure 6) and CaR biofilms (Figure 7). In contrast, the group treated with nystatin alone neither prevented the penetration of *C. albicans* into the 3D coculture tissues nor reduced the fungal load.

Infection by *C. albicans* is associated with host cell damage.^51^ In mucosal infection, the interaction between epithelial cells and fungal components leads to induced endocytosis and active penetration by *C. albicans*.^52,53^ During this process, *C. albicans* secretes hydrolytic enzymes that affect epithelial cell–cell junctions and facilitate degradation of cell membrane components.^54^ Other studies describe specific mechanisms, such as the secretion of the Candidalysin toxin by *C. albicans* hyphae, which causes cell lysis and significantly contributes to tissue damage and inflammatory responses.^51^ In the present study, epithelial cell damage induced by *C. albicans* in the 3D coculture model was assessed through the quantification of LDH release.^55^ It was observed that the level of LDH release in the infected control group and death control group was substantial. On the other hand, the isolated application of ZER, regardless of concentration, significantly reduced LDH release, despite decreasing the fungal load by approximately 1 log_10_, suggesting protective effect of ZER on the tissue. In addition, the combination of ZER with aPDI decreased the cell damage in the 3D coculture model infected with *C. albicans*, as indicated by the amount of LDH release (7.41% in CaS and 9.43% in CaR), with levels comparable to those of the noninfected control. On the other hand, the NYS group showed high levels of LDH (32% for CaS and 42% for CaR) compared to the ZER + aPDI group. The group exposed exclusively to light exhibited elevated LDH release (69.31% for CaS and 48.50% for CaR). This behavior can be attributed to the photoexcitation of endogenous porphyrins, which triggers ROS production and alters membrane permeability in both fungal and host cells.^56^ On the other hand, the association of ZER with light (ZER + Light) resulted in reduced LDH expression levels compared to the infected control. This finding suggests that ZER may exert a cytoprotective effect, mitigating the oxidative stress and membrane leakage observed when light is used alone.^57^ These findings corroborate a previous study that observed that the ZER group showed decreased LDH levels compared with the zearalenone group (ZEA, a mycotoxin mainly produced by *Fusarium* species), and these results indicate that ZER pretreatment protects the liver and can inhibit ZEA-induced hepatotoxicity.^58^ Similarly, Yang et al.^59^ demonstrated potent protective effects of ZER in dermal terms against UVA-induced cellular damage. In both UVA-irradiated human keratinocytes (HaCaT cells) and mouse epidermis, pretreatment with ZER (2–10 μM) significantly mitigated UVA (15 J/cm^2^)-induced cell death and LDH release in a dose-dependent manner.^59^ ZER effectively counteracted key UVA-mediated cytotoxic events, including the excessive generation of ROS, DNA single-strand breaks, apoptotic DNA fragmentation, and dysregulation of the Bax/Bcl-2 ratio.^59^ Collectively, these findings highlight the protective role of ZER in preventing UVA-induced photooxidative damage and enhancing cellular antioxidant defense mechanisms.^60^ ZER also inhibited intracellular oxidative stress and apoptosis induced by mycotoxin in the liver tissue of albino mice by activating the PI3K/AKT-mediated Nrf2/HO-1 signaling pathway.^61^ Therefore, ZER demonstrates potential effect as a therapeutic agent for the prevention of hepatic damage.^58^ These antioxidant and protective properties may also have contributed to the effects observed in our 3D coculture infection model, likely minimizing epithelial cell damage by attenuating the oxidative stress induced during *C. albicans* infection.

Infections caused by *C. albicans* modulate the host inflammatory response, leading to increased expression of proinflammatory interleukins such as IL-6, IL-1β, and IL-8. It was reported that proinflammatory cytokine production (IL-6 and IL-8) was upregulated in epithelial cells following infection by *C. albicans*.^52^ This upregulation reflects the activation of host tissue cells in response to the infection process.^28,29^ In agreement, the present study demonstrated that 3D cocultures infected with *C. albicans* also exhibited increased production of IL-6 and IL-8, further supporting the validity of the 3D coculture model employed. The elevated LDH release was associated with high levels of IL-6 and IL-8, indicating enhanced proinflammatory cytokine production during *C. albicans* infection. These elevated levels of IL-6 and IL-8 may result from cytokine release triggered by cell lysis and activation of the damage-associated receptors. However, other key cytokines, such as TNF, IL-1β, and IL-10, were not detected, probably due to the nature of the 3D epithelial model employed here, which, despite its structural complexity, lacks a functional immune component (e.g., macrophages and T-cells) responsible for the primary secretion of these specific mediators.^27,28,58^ Furthermore, the treatment of the 3D coculture model with ZER (1172 μM) followed by aPDI resulted in a substantial reduction (>90%) in IL-6 and IL-8 levels. Therefore, the combination of treatment was effective against *C. albicans* biofilms and may hinder further infection progression and subsequent tissue damage.

Studies have demonstrated that ZER exerts its anti-inflammatory effects through the modulation of multiple cellular signaling pathways.^61^ The literature indicates that inhibition of the NF-κB pathway represents a key mechanism, as this pathway acts as a central regulator of proinflammatory gene expression.^30^ By suppressing NF-κB activation, ZER may contribute to reducing the production of key proinflammatory cytokines, such as IL-6 and IL-8, which may help explain the effects observed in the *C. albicans*-infected 3D coculture model in the present study. Additional mechanisms may include the induction of heme oxygenase-1 (HO-1), an enzyme with well-established anti-inflammatory and antioxidant properties,^62^ as well as the inhibition of nitric oxide and prostaglandin E2 production in lipopolysaccharide-stimulated macrophages.^63^ It was demonstrated that ZER was able to inhibit proinflammatory cytokines induced by ZEA, a mycotoxin mainly produced by *Fusarium graminearum* and *Fusarium culmorum.* ^58^ In that study, by suppressing the transcriptional activation of p65, ZER reduced the expression of inflammatory enzymes and cytokines, thereby protecting hepatic tissue against ZEA-induced oxidative stress and cellular damage.^58^ These anti-inflammatory properties, along with the observed low cytotoxicity, reinforce the therapeutic potential of ZER in combination with aPDI as a multifaceted strategy for treating oral fungal infections. This combined approach not only targets the pathogen but may also modulate the detrimental host inflammatory response.

In conclusion, the outcomes of this study showed the biocompatibility and efficacy of ZER in combination with aPDI for controlling fluconazole-sensitive and -resistant *C. albicans* biofilms in a 3D coculture model. The synergistic interaction between ZER and aPDI in a 3D coculture model significantly reduced fungal load and biofilm penetration, outperforming either treatment alone. This combination may represent a promising strategy for combating resistant fungal infections and offer a potential alternative to conventional antifungal therapies. Furthermore, this study reinforces the utility of 3D coculture models for accurately assessing the biocompatibility and antimicrobial efficacy of novel therapeutic approaches, offering a more physiologically relevant platform for preclinical studies. Although the results are promising, translation to the clinic requires further preclinical investigation to validate potential treatment protocols before they can be applied in clinical studies. We suggest further research to investigate the additive and synergistic potential of ZER with other photosensitizers available for aPDI as well as to establish its safety and efficacy in multispecies biofilm infection models.

## Abbreviations

aPDI: Antimicrobial Photodynamic Inactivation
ANOVA: analysis of variance
ATCC: American Type Culture Collection
CaR: *Candida albicans* fluconazole-resistant
CaS: *Candida albicans* fluconazole-sensitive
CFU/mL: colony-forming units per milliliter
CSLM: Confocal Laser Scanning Microscopy
DMEM: Dulbecco’s Modified Eagle’s Medium
ECM: extracellular matrix
FBS: fetal bovine serum
HGF: Human Gingival Fibroblast
IL: interleukins
LDH: Lactate Dehydrogenase
PI: Propidium Iodide
PDZ: Photodithazine
RPMI: Roswell Park Memorial Institute
ROS: reactive oxygen species
SDA: Sabouraud Dextrose Agar
YNB: Yeast Nitrogen Base
ZER: Zerumbone
2D: two-dimensional
3D: three-dimensional
